# Phosphorus and Nitrogen Modulate Plant Performance in Shrubby Legumes from the Iberian Peninsula

**DOI:** 10.3390/plants8090334

**Published:** 2019-09-06

**Authors:** María Pérez-Fernández, Ángel Míguez-Montero, Alexandre Valentine

**Affiliations:** 1 Department of Physical, Chemical and Natural Systems, University Pablo de Olavide, 41013 Seville, Spain; 2 Department of Botany and Zoology, Stellenbosch University, 7602 Matieland, South Africa

**Keywords:** phosphate, nitrogen, drought, *Genistea*, nutrients use efficiency

## Abstract

We investigated the impact of phosphorus nutrition on plant growth and biological nitrogen fixation in four leguminous plants in the Tribe *Genistea*. The main objective of the study was to analyze Phosphorus and Nitrogen use efficiency under drought. We also tested for the effects of rhizobial inoculation on plant performance. Plants inoculated with *Rhizobium* strains isolated from plants of the four species growing in the wild were cropped under controlled conditions in soils with either low P (5 µM) or high P (500 µM). The experiment was replicated in the presence and absence of plant irrigation to test for the effects of drought stress of inoculated and non-inoculated plants under the two P levels of fertilization. Low-P treatments increased nodule production while plant biomass and shoot and root P and N contents where maximum at high P. Low P (5 µM) in the growing media, resulted in greater N accumulated in plants, coupled with greater phosphorus and nitrogen uptake efficiencies. Drought reduced the relative growth rate over two orders of magnitude or more, depending on the combination of plant species and treatment. *Genista cinerea* had the lowest tolerance to water scarcity, whereas *Genista florida* and *Retama sphaerocarpa* were the most resistant species to drought. Drought resistance was enhanced in the inoculated plants. In the four species, and particularly in *Echinospartum barnadesii*, the inoculation treatment clearly triggered N use efficiency, whereas P use efficiency was greater in the non-inoculated irrigated plants. Nodulation significantly increased in plants in the low P treatments, where plants showed a greater demand for N. The physiological basis for the four species being able to maintain their growth at low P levels and to respond to the greater P supply, is through balanced acquisition of P and N to meet the plants’ nutritional needs.

## 1. Introduction

Around 45% of the earth’s surface covers arid and semi-arid areas [1]. The Mediterranean Basin is part of this semi-arid land, with reduced availability of nutrients like nitrogen and phosphorus. Lack of nutrients, together with limited rainfall, hinder plant performance [2]. In the center of Spain, where harsh environmental conditions are predominant, wild shrubby legumes are key in the maintenance of natural ecosystems. Shrubby legumes prevent erosion, create islands of fertility, and facilitate the establishment of other plant species at the time that they contribute to soil stabilization and improvement of ecosystems through N_2_ fixation [3,4]. In the Iberian Peninsula, shrubby legumes are widely distributed and where trees are absent, they maintain active ecosystems thanks to their nitrogen-fixing ability. Shrubby legumes have a high N_2_-fixing potential that makes them an important component of sustainability and soil fertility [4,5].

Current global change is disrupting the equilibrium of natural ecosystems in the Iberian Peninsula. The decline in soil and plant productivity, particularly in the Iberian soils subjected to moisture deficiency, leads to an irreversible situation of desertification. In addition, overgrazing, deforestation and non-regulated cultivation in natural systems are accelerating the soil degradation problem [6]. The result is a dominant ecosystem with disturbed or lost vegetation cover, increased soil erosion, depletion of organic matter and nutrients, breakdown of the biogeochemical cycles, and a reduction in soil microbial activities [6,7]. The final outcome is a decline in plant productivity followed by extensive and continued soil degradation. The establishment and/or maintenance of a suitable plant cover is known to improve the chemical, physical, and biological properties of the soil [4,6,7] as the formation of a dynamic rhizosphere is critical for the well-functioning of ecosystems, particularly in dry, low-nutrient ecosystems [7].

Shrubby legumes are key in the re-vegetation of water-stressed ecosystems characterized by low availability of N and P and other nutrients, as they undergo beneficial symbiosis with rhizobia. In the Iberian Peninsula, there is a large number of shrubby legumes that are nodulated by a diverse group of rhizobia (*Rhizobium* and *Bradyrhizobium*). Despite the fact of this richness, only a few studies have characterized the diversity of rhizobia associated with these native legumes [2,8]. There is little information in regards to the selection of microsymbionts to maximize the biological N_2_ fixation under stressful environments and to use them as key elements in the recovery of degraded soils and to maintain active natural ecosystems. The potential of these root nodule bacteria as symbionts with their host legumes has not yet been fully explored.

Phosphorus is one of the most limiting nutrients for plant growth [9,10]. Such scarcity of available P and the imbalance of trace elements in the semi-arid ecosystems of Iberian Peninsula actually limit legume establishment and N_2_ fixation. In fact, the addition of P to plants growing in poor soils significantly increases plant nodulation and biomass production [9,10,11]. Nitrogen fixation under P deficiency is often reduced, because low P supply induces changes in the relative growth of nodules and shoots [12]. Nodules require relatively higher amounts of P and energy than do other plant tissues [13]. The response of nodulation and N_2_ fixation to water stress depends on the growth stage of the plants and on the plant’s nutritional stage. Nodule P concentrations and P use efficiency are positively correlated with soil and root water content in symbiosis where *Bradyrhizobium* is present [14]. Similarly, N derived from N_2_ fixation decreases under water deficiency [15]. The broad responses of plant-rhizobia to moisture levels suggest that rhizobial strains show different sensitivity to soil moisture as well as to N and P levels [16,17]. Drought-tolerant, N_2_-fixing legumes, and legumes tolerant to low levels of nutrients in the soil can be selected to grow in arid regions [18,19,20] as they can aid plants to grow at the time that improves soil fertility in arid and semiarid habitats.

In this study, we aimed to evaluate the response of four shrubby leguminous plant species native to the Iberian Peninsula, to two water regimes and to two levels of P in the growing media in the presence and absence of their rhizobial symbionts. The main objective of this study was to assess the importance and effectiveness of legume–rhizobia symbioses as a tool in the management of natural ecosystems through their introduction for re-vegetation and for the maintenance of healthy ecosystems. Analyses of plant tolerance to stress were measured through nodule production, plant biomass accumulation, and N and P accumulation in plant material, as well as the rates of N and P uptake and N and P use efficiency. We hypothesize that when P availability is restricted, the presence of nodule-forming and N-fixing bacteria confer an advantage to plants that are better able to cope with nutrient scarcity. We relate the ability of plants to cope with P and water scarcity to their natural distribution in nature. To that end, we chose four shrubby legumes from the Iberian Peninsula that naturally grow in poor and dry environments and propose that the rhizobia–legume symbiosis is more efficient in plants originating in poor soils and with less water availability, than plants originating in milder environments.

## 2. Materials and Methods

### 2.1. Plant Culture and Growth Conditions

Seeds of *Echinospartum barnadesii* sbsp *dorsisericeum* G.López, *Genista cinerea* DC, *Genista florida* L., and *Retama sphaerocarpa* (L.) Boiss., were hand harvested in natural populations of the four species, and preserved in brown envelopes at 14 ± 1 °C till used in the laboratory. Selection of the species was based on their global distribution, *E. barnadesii* and *G. florida* are endemic to the Iberian Peninsula (i.e., restricted distribution) and both require acidic and dry soils; whereas *G. cinerea* and *R. sphaerocarpa* have a wider distribution along Africa and Europe. The former has a basic preference for soil, whereas the latter is edaphically indifferent [21].

Prior to germination, seeds were surface sterilized by immersion in 98% ethanol for 1 min, 4% sodium hypochlorite for 4 min and rinsed with sterile distilled water. Germination was conducted in petri dishes containing plain agar. Thirty seedlings (1-week old) per species and treatment were transplanted individually to plastic tubes (80 mm diameter, 160 mm long) containing sterile sand-river and were given the appropriate treatment. All seedlings were supplied with 25% Hoagland’s solution, pH 5.3 [22], modified with either high P (500 µM) or low P (5 µ M) as NaH_2_ PO_4_ 2H2O. Plants were maintained in a glasshouse at the University Pablo de Olavide (Seville, Spain) under natural light regime and temperature, with a 12-h photoperiod (24 °C day and 18 °C night) and a photon flux density at the top of the plants of approximately 700 µmol m^−2^.s^−1^ for 36 weeks. Pots with different treatments were randomly distributed on benches in the glass house, 1 m apart from any other treatment, to prevent cross contamination; a total of 30 replicates per combination of species and treatments were maintained. The control treatment consisted of un-inoculated Hoagland’s solution from which nitrogenous compounds had been removed. One of the treatments consisted of nitrogen-free Hoagland’s solution and rhizobial inoculation (Inoculation). A second treatment consisted of the application of 500 µM NaH_2_ PO_4_ 2H_2_O (highP) with no rhizobial inoculation. In the third experiment, plants received the same amount of NaH_2_ PO_4_ 2H_2_O as before and were simultaneously inoculated. In the fourth treatment, plants received 5 µM NaH_2_ PO_4_ 2H_2_O (low P) with no rhizobial inoculation. In the last experiment, plants received the same amount of NaH_2_ PO_4_ 2H_2_O as before and were simultaneously inoculated.

Inoculants consisted of a heavy suspension of the log-phase culture on yeast mannitol broth, pH 6.8, at 1 × 10^7^ cells mL^−1^. Seedlings were inoculated with the appropriate *Bradyrhizobia* previously extracted from nodules from adult plants in wild populations. All inoculants had been reported to be *Bradyrhizobium* and their accession numbers are AF443639 for *E. barnadesii*, Z72261 for *G. cinerea*, Z72265 for *G. florida* and AF46119 for *R. spahaerocarpa* [8]. Soil contamination during experimentation was avoided by placing each pot in individual plastic bags, and every pot was covered with a layer of autoclaved plastic beads of 0.5 mm diameter (Industrial Aulabor S.A., Barcelona, Spain). Watering was performed through an autoclaved watering pipe.

For every species, each combination of P level and inoculation received two levels of irrigation. In the first treatment, pots were maintained at 70% soil field capacity, resembling the growing conditions of the species in spring in their natural habitats. In the second treatment, soils were maintained at 35% soil field capacity, representing a stressful situation of drought. Field capacity was estimated prior to the experiment by placing a saturated known amount of soil in a pot covered by thin plastic film that was allowed to drain overnight. After that, the soil was weighed. The same soil sample was oven dried at 72 °C till constant weight. One-hundred percent field capacity was estimated as the water held by the soil and subsequently calculated water held at 70% and 35% field capacity [23]. Four random tensiometers were placed in pots of each of the treatments to measure soil field capacity in the pots daily (Nielsen et al. 1964). Seedlings were supplied with sterile distilled water when needed to maintain the desired percentages of field capacity in the pots and to maintain the initial concentration of P applied in the described experiments. The experiment was split among the four species, so that each species was subjected to low- and high P treatments, water and drought. For every combination of P and water treatment, with and without inoculation, there were 30 plants in each of the four species. Plants were harvested after 18 and 36 weeks of growth. The number of nodules and active nodules per plant, shoot and root dry weights (72 h at 75 °C) were recorded.

### 2.2. Nitrogen and Phosphorus Efficiencies and Allocation Calculations

Shoot and root dry matter was ground to pass a 20-mesh sieve and digested with a mixture of H_2_SO_4_–H_2_O_2_ [24]. Total nitrogen was analyzed by the Kjeldahl method [25] and the phosphorus content was determined colorimetrically on digested samples with a solution of perchloric acid (72%), ammonium molybdate (5%, aminonaphtholsulphonic acid (0–2 %), 0.5 g of sodium bisulphite and 6 g crystalline sodium sulphite [25]. Whole plant nutrient content in each plant was calculated by multiplying each nutrient concentration by its respective dry biomass value.

Phosphorus and Nitrogen uptake efficiency (PUpE and NUpE) and physiological P and N use efficiency (PPUE and PNUE) were calculated for each of the species using equations adapted from [26] as:

(1) $$\mathrm{PUpE}\;\left(\mathrm{mgP}\;g\cdot {\mathrm{Kg}}^{-1}\mathrm{Ps}\right)=\frac{\left(\mathrm{PHigh}\times Y\;\mathrm{High}\right)-\left(\mathrm{PLow}\times \mathrm{YLow}\right)}{\Delta \mathrm{Ps}}$$

(2) $$\mathrm{NUpE}\;\left(\mathrm{mgP}\;g\cdot {\mathrm{Kg}}^{-1}\mathrm{Ps}\right)=\frac{\left(\mathrm{NHigh}\times Y\;\mathrm{High}\right)-\left(\mathrm{NLow}\times \mathrm{YLow}\right)}{\Delta \mathrm{Ns}}$$

(3) $$\mathrm{PUE}\;\left(g^{2}\cdot \mathrm{DW}\cdot {\mathrm{mg}}^{-1}\cdot P\right)=\frac{\left(\mathrm{YLow}\right)}{\mathrm{PLow}}\mathrm{or}\frac{\left(\mathrm{YHigh}\right)}{\mathrm{PHigh}}$$

where PHigh and NHigh, and PLow and NLow were the whole plant [P] and [N] (mg/g plant) in plants grown in the soil with high and low contents of P; YHigh and YLow were the total plant biomass (g) in the treatments with high and low contents of P. All treatments were replicated for irrigated and water-stressed plants, both in the high- and low P content treatments.

### 2.3. Below Ground Allocation

Belowground allocation represents the fraction of new biomass partitioned into new roots and nodules over the total growth period. The calculations were performed according to [27]:

df/dt = RGR (∂ − Br/Bt)(4)

where RGR is the relative growth rate (mg·g^−1^·day^−1^) and ∂ is the fraction of new biomass gained during the growth period. Br/Bt is the root weight ratio, based on total plant biomass (Bt) and root biomass (Br).

### 2.4. Statistical Design and Analyses

The experimental design was a complete randomized block design. The growth values and parameters relating to N and P concentrations were means of 10 to 15 replicates per treatment, depending on plant survivorship. The effects of the factors and their interactions were tested with two-way analysis of variance (ANOVA). Where the ANOVA revealed significant differences, a Student Newman Kuehl’s (SNK) multiple-range test (* *p* < 0.05) post-hoc test was calculated. Different letters indicate significant differences between treatments. All samples were normal, and homogeneous variances were tested using the Levene and Cochran tests implemented on SPSS 21.0 software.

## 3. Results

### 3.1. Plant Survivorship

**Figure 1:**
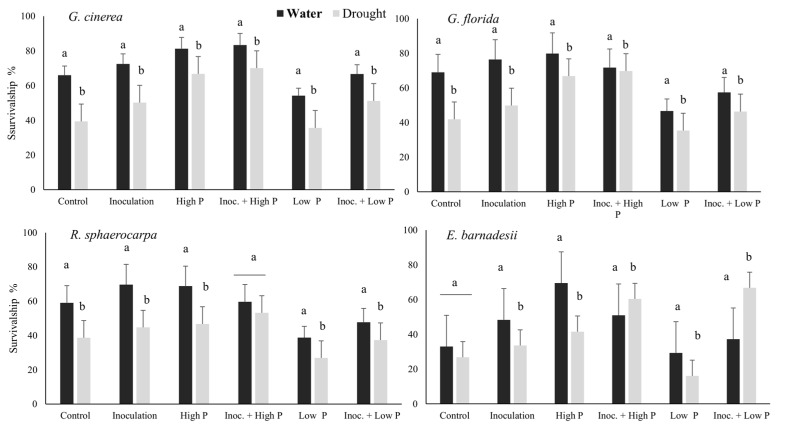
Survivorship of plants of *G. cinerea*, *G. florida*, *E. barnadesii* and *R. sphaerocarpa* grown under two P treatments (500 µM and 5 µM) with and without inoculation in well irrigated and drought-stress tests. Treatments were control, inoculation, high P, inoculation and high P, low P, inoculation and low P in water and drought condition.

Percentages of plant survivorship ranked between 18% in water stress *E. barnadesii* plants grown at low P and 83% in well irrigated *G. cinerea* plants grown inoculated under high P (Figure 1). Survivorship of *Genista florida*, *R. sphaerocarpa* and *E. barnadesii* peaked in the high P treatment whereas *G. cinerea* did so at high P inoculated plants. Drought treatment induced a significant reduction in survivorship in all species but *E. barnadessi* grown inoculated both high and low P (Figure 1).

### 3.2. Biomass Production and Growth Kinetics

**Figure 2:**
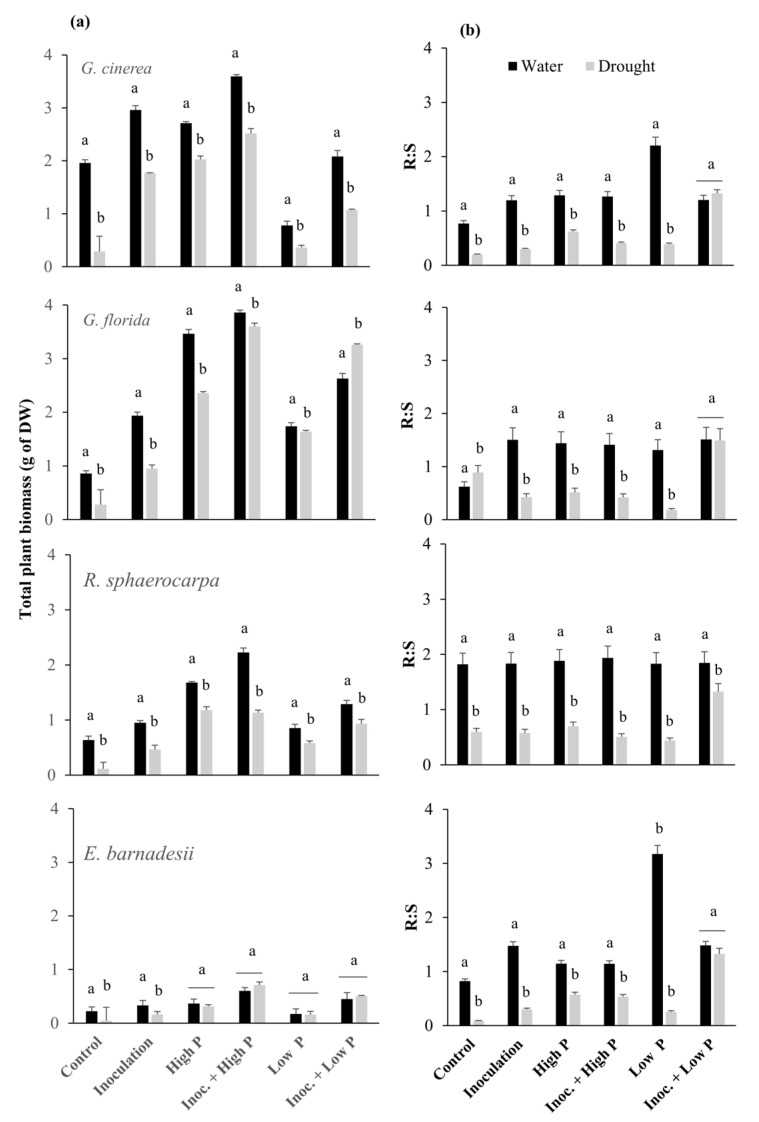
Plant dry weights (**a**) and shoot:root ratio (**b**) of *G. cinerea*, *G. florida*, *E. barnadesii* and *R. sphaerocarpa* after receiving either 500 μM P (High) or 5 μM P (Low) nutrient solutions with and without bacterial inoculation, under two water regimes: Irrigation (Water) and drought stress (drought). Values are presented as means (*n* = 15) with standard error bars and letters indicating significant differences among treatments using the post-hoc Fisher’s LSD, multiple range test (*p* ≤ 0.05).

Total plant biomass of *R. sphaerocarpa* in any of the treatments was half of that produced by any of the two *Genista* species (Figure 2a). Plant biomass production of *E. barnadesii* was one-tenth of that of the other species. *G. cinera*, *G. florida* and *R. sphaerocarpa* produced more biomass in the well-irrigated treatments whereas *E. barnadesii* had its biomass production increased in the Inoc+high P, low P and Inoc+low P treatments under drought conditions. Inoculation significantly increased the biomass production in the four species both in the well irrigated and drought treatments compared with the control (Figure 2a).

Well irrigated plants of *E. barnadesii* and *G. cinerea* in the low P treatment showed the greatest root production. Root production in *R. sphaerocarpa* was homogeneous in the well-irrigated plants regardless of the P treatments (Figure 2b). Plants produced more below ground biomass in the well-irrigated treatments, except for plants of *G. cinenera*, *G. florida* and *E. barnadesii* in the Inoc+low P treatments, where no differences were observed between watered and drought treatments.

Growth rate was significantly reduced in the drought treatment in the four species (Table 1). Water stress resulted in biomass partition in plant organs. Root growth was not significantly affected by drought, while there was a major decline in shoot production as seen in the lower root:shoot ratio (Figure 2). *G. cinerea* and *E. barnadesii* under the two water regimes had their RGR increased under the Inoculation and high P + Inoculation treatments. *G. florida* and *R. sphaerocarpa* were mostly enhanced in their RGR by the inoculation regardless of the level of P applied and the water regime. In addition, RGR of *G. florida* was also significantly enhanced by the high P in the drought treatment.

**Figure 3:**
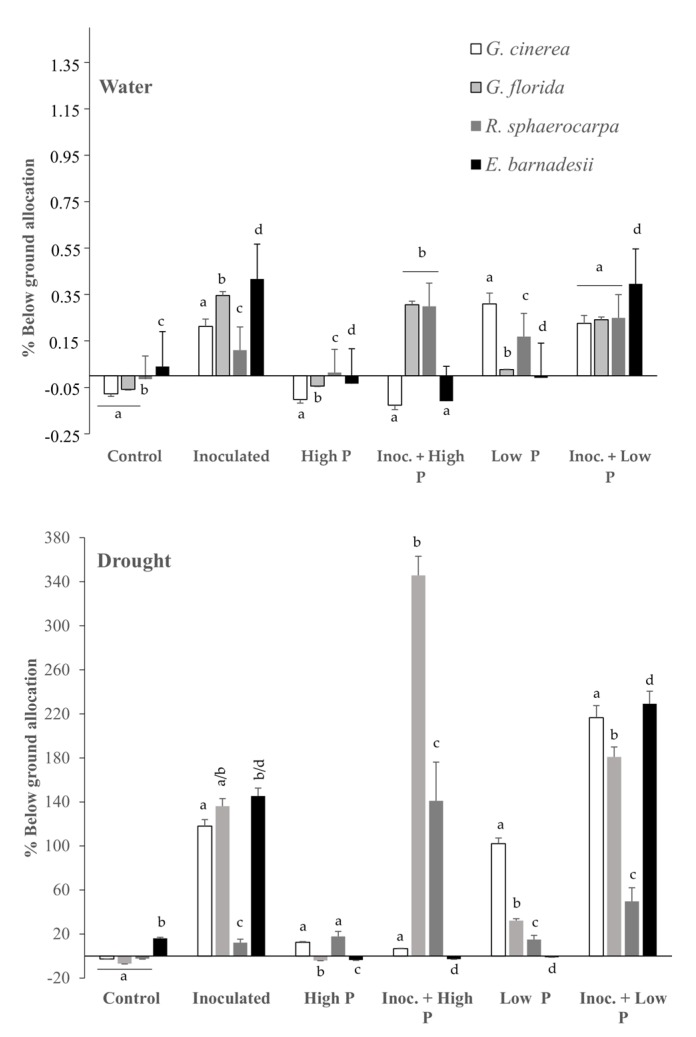
Percentage (%) of below-ground allocation of roots of *G. cinerea*, *G. florida*, *E. barnadesii* and *R. sphaerocarpa* plants supplied with high (500 µM) and low (5 µM) phosphorus, inoculated and non-inoculated under two water regimes (water and drought stressed). Values are presented as means (*n* = 11–15) with standard error bars. The different letters indicate significant differences among the treatments (*p* ≤ 0.05).

Plants allocated more of their resources to roots in the drought stress treatments than in the water ones (Figure 3). The inoculation treatment clearly enhanced root allocation under the two water regimes, with *R. sphaerocarpa* being the species with the lowest root mass production. Under high P, only *R. sphaerocarpa* increased its root allocation, although no significant differences were detected. However, the treatment of Inoc + low P in the four species was the one that induced the greatest root biomass production, although this positive effect was not statistically significant in *R. sphaerocarpa*. This latter species, together with *G. florida*, enhanced their root production under the Inoc + high P treatment. Low P significantly induced root allocation in *G. cinerea*, whereas high P did not have the same positive effect (Figure 3).

### 3.3. Nodule Formation and Infection

**Figure 4:**
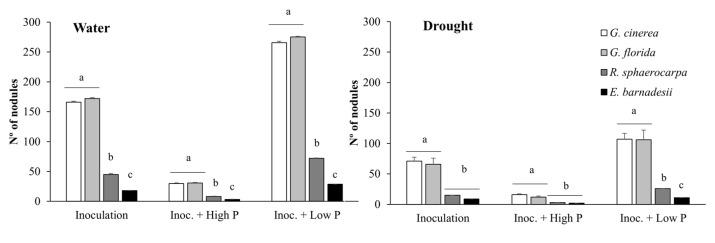
Number of nodules per plant of *G. cinerea*, *G. florida*, *E. barnadesii* and *R. sphaerocarpa* grown inoculated and inoculated plus high (500 µM) or low (5 µM) Phosphorus, under two water regimes (water: well irrigated; drought: water stress). Values are presented as means (*n* = 13–15) with standard error bars and letters indicating significance.

No nodulation was observed in the non-inoculated plants, proving that there was no bacterial contamination. The four species grown under high P treatment significantly decreased nodule production, compared with the other treatments (Figure 4). Seedlings in the control inoculated treatment and in the Inoc + low P produced significantly more nodules than in the rest of the treatments. In both the control and Inoc + low P, there were significant differences in nodules production between the water and drought treatments (*p* = 0.0214). *R. sphaerocarpa* and *E. barnadesii* produced significantly less nodules than the other two species regardless the treatment (Figure 4).

### 3.4. Nitrogen and Phosphorus Efficiencies and Allocation

**Figure 5:**
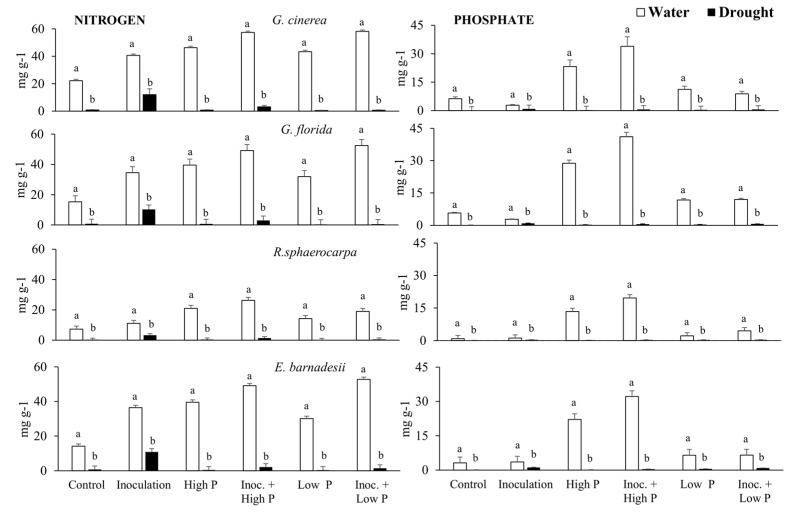
Nitrogen and phosphorus content in *G. cinerea*, *G. florida*, *E. barnadesii* and *R. sphaerocarpa* grown under six treatments of inoculation and P sources: control, inoculation, high Phosphorus, inoculation and high phosphorus, low Phosphorus, inoculation and low phosphorus in water condition and drought conditions. Values are presented as means (*n* = 13–15) with standard error bars. Statistical analyses were performed independently for N and for P. Different letters on top of columns indicate significant differences for each nutrient across treatments (drought and water) using the post-hoc Fisher’s LSD, multiple range test (*p* ≤ 0.05).

Total P concentration in well-irrigated plants was very low in seedlings of *R. sphaerocarpa*. P concentration for well-irrigated plants of the four species was always significantly greater in the high P treatment (Figure 5). The accumulation of P in seedlings of the four species in the drought experiment was negligible. In the water experiment, *R. sphaerocarpa* accumulated less N than plants of *G. cinerea.* Whereas *G. florida* and *E. barnadesi* accumulated similar amounts of N (Figure 5). N accumulation in the four species followed a similar pattern, with the greatest N accumulation in the Inoc + high P and high P treatments, followed by Inoc + low P. In the drought treatments, N accumulation was maximum in the Inoculation treatment in the four species followed by Inoculation + high P, although values were significantly lower than those observed in the well-irrigated plants.

**Figure 6:**
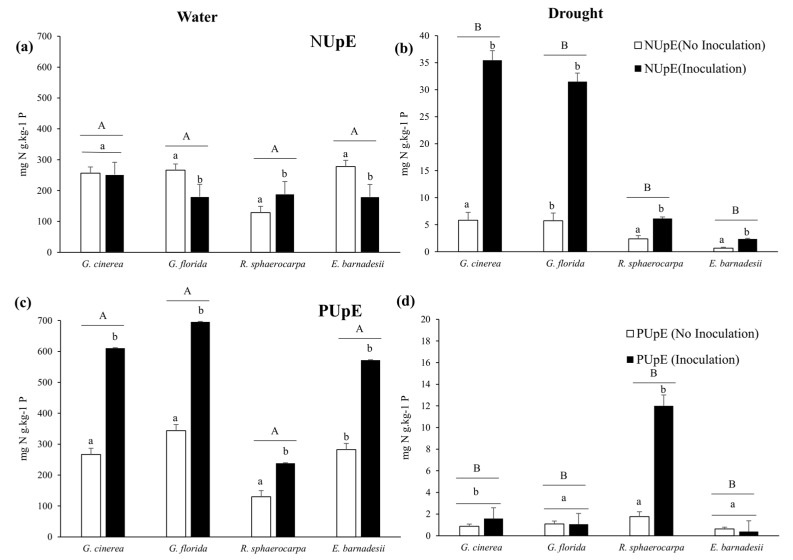
Nitrogen and Phosphorus uptake efficiencies (NUpE and PUpE) of *G. cinerea*, *G. florida*, *E. barnadesii* and *R. sphaerocarpa* after receiving either 500 µM (High P) or 5 µM (Low P) nutrient solutions with and without rhizobial inoculations under two water regimes (water: well-irrigated plants—left graphs; drought: water stressed plants—right graphs). Values are presented as means (*n* = 13–15) with standard error bars. Upper case letters indicate significant differences between water treatments, and lower case letters indicate significant differences among inoculation treatments using the post-hoc Fisher’s LSD, multiple range test (*p* ≤ 0.05). Note different scales.

The decline in these elements in the water stress plants coincided with reduced levels of uptake efficiency (NUpE and PUpE) in the four species when plants were not inoculated. However, NUpE in the well irrigated-non inoculated plants was significantly greater in *G. frorida* and *E. barnadesii* compared with the rest of the treatments. Similarly, NUpE in the well-irrigated non-inoculated *R. sphaerocarpa* plants was significantly lower compared with the remaining treatments. Finally, no differences were observed in plants of *G. cinerea* (Figure 6). There was a clear significant increase in the NUpE and PUpE in the inoculated plants, both in the watered and drought experiments (Figure 6). PUpE was significantly reduced in the non-inoculated plants of the four species in both the well-irrigated and drought-stress plants.

**Figure 7:**
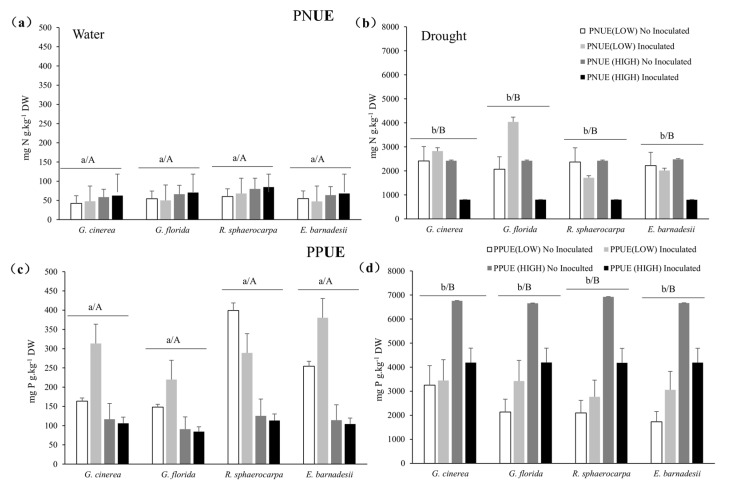
Nitrogen and Phosphorus physiological use efficiencies (PNUE and PPUE) of *G. cinerea*, *G. florida*, *E. barnadesii* and *R. sphaerocarpa* after receiving either 500 µM (High P) or 5 µM (Low P) nutrient solutions with and without rhizobial inoculations under two water regimes (water: well-irrigated plants (**a**,**c**); drought: water-stressed plants (**b**,**d**). Values are presented as means (*n* = 13–15) with standard error bars. Upper case letters indicate significant differences between water treatments and lower case letters indicate significant differences among inoculation treatments using the post-hoc Fisher’s LSD, multiple range test (*p* ≤ 0.05).

Physiological N use efficiency (PNUE) was significantly greater in the drought stress plants in the four species (Figure 7a,b). No differences among treatments were observed in the water treatment, regardless of the level of P and the inoculation treatment. In the water stress plants of *G. cinerea* and *G. florida*, inoculation at low P significantly enhanced PNUE, whereas inoculation of high P treated plants, reduced PNUE. In the four species, the well irrigated plants Inoc + low P showed significantly high values of PPUE. Only in *R. sphaerocarpa,* PPUE was greater in the low P treated plants, compared with the rest of the treatments (Figure 7c,d). PPUE in the water-stress plants (Figure 7d) followed a similar pattern in the four species, with significantly higher PPUE values under the high P and high P + Inoculation, compared with the rest of the treatments.

## 4. Discussion

Water availability clearly determines plant survivorship and biomass production in the four species as well as relative growth rate. Similarly, mineral nutrition is also crucial in plant matter production as the four species perform better when grown at high levels for P. This biomass production is even greater when plants are inoculated compared with the un-inoculated controls (Figure 2). *G. cinerea* and *G. florida* were the two species that benefited the most from inoculation. Both species cope with water scarcity by increasing their root allocation, although the amount of biomass devoted to roots clearly depends on the levels of P in the soil. This strategy is shared by *R. sphaerocarpa* and *E. barnadessii* in this study, and other shrubby legumes in soils of reduced fertility [10,28,29]. *G. florida* and *E. barnadesii* have their natural range of distribution on acidic soils in arid areas, where nutrients like P are more unavailable than in basic soils [30]. To meet their nutritional requirements, plants have to explore a broader area of soil that can only be achieved at the expense of a greater belowground allocation (Figure 3) [31]. *R. sphaerocarpa* is recognized not to be strongly affected by drought due to its tap root, that can access water in almost any kind of soil [32]. In this sense, it is not surprising that this species modifies biomass allocation to either roots or shoots in varying environments and in view of the mineral nutrition available. *E. barnadesii* usually grows in very acidic soils with low levels of nutrients. In this study, variation in below-ground allocation is related to nutrient availability, with reduced allocation to roots when nutrients are abundant in the soil, a strategy shared with other species in the *Genistea* Tribe [10,33].

Nodulation was clearly more efficient in the well-irrigated plants than in the stressed ones (Figure 4 and Figure 5). In the four species, in the water treatment, the greatest number of nodules was observed in the Inoc + low P treatment and the highest N accumulation occurred in the Inoc + high P plants (Figure 5), which coincides with a peak in plant biomass production in the four species (Figure 1). However, in the water-stressed plants, N and P accumulation did not differ between Inoc + high P and Inoc + low P and that can only be explained by a more efficient nodulation in the water-stressed plants following the same pattern as in other leguminous species [4,34,35,36,37]. This behavior has been already described in other species in the Genistea family under several stresses [38,39]. We have also observed a synergistic effect of P addition and inoculation in plant growth, as the relative growth rate under this treatment was the highest in the four species. *G. cinerea and E. barnadesii,* the two species from very poor and acidic soils, responded better to the combination of inoculation and low P, whereas *G. florida* and *R. sphaerocarpa*, naturally growing in basic soils, had better responses to the combined inoculation with high P. The former species have the ability to acquire P from soil and use it efficiently, increasing plant performance in the short term and even when P is present in a very low concentration. This can be understood as an evolutionary response to the acidic soils where these plants grow, as has been observed in other legumes [4,10,34,37,40]. The other two species, *G. florida* and *R. sphaerocarpa*, whose distribution in natural systems corresponds with richer soils, show lower values of relative growth and do not show signs of need for a quick mineral nutrition acquisition [41].

We did not measure actual Biological Nitrogen Fixation (BNF). Nevertheless, BNF happened as demonstrated by the greater N accumulation in inoculated plants compared with the non-inoculated ones. In addition, there was no mineral N added to any of the treatments, for which the afore-mentioned greater accumulation of N in inoculated plants can only be attributed to the effective nodulation and BNF, as stated in other studies, where BNF is measured by comparing total N in control reference plants and inoculated ones [42,43]. BNF is a very expensive process for the legume; when P and N are sufficient in the soil, plants prefer these mineral nutrients, avoiding the expensive biological fixation. In our study, under high P conditions, the four species (Figure 5) did accumulate more P in both the high P and the Inoc + high P treatments, thus avoiding expenses in BNF [34,44]. Contrary to that, in the control and low P treatments, plants produced very efficient nodules, resulting in greater nitrogen accumulation in the whole plant. In fact, the root allocation of nodulated plants is the highest. This proves the requirement for the plants to maintain all the structures that guarantee N acquisition as it has been observed in *Virgilia divaricata* [5] and other legumes from nutrient poor ecosystems, where the belowground organs have a higher resource allocation under low P values [34,45]. The BNF in legumes is a costly process, very much dependent on phosphate supply. When P is limiting, nodule formation and the nitrogen fixation process is altered [37,46]. However, under P deficiency, some legumes can shift their nitrogen assimilation process, being forced to acquire N through symbiotic N_2_ fixation [9,41], a fact commonly observed in other shrubby legumes form the Iberian Peninsula [29].

The ability to acquire nutrients from the soil and use them efficiently for biomass production is an important characteristic for plant adaptation to soils low in them [47]. The highest NUpE, a measure of the aggregate effect of nutrients acquisition mechanisms, was observed in non-inoculated plants in the water treatment. This result is in accordance with the maximum N accumulation in plants, which is in line with their high root:shoot ratios. When water is not limiting, and there is a source of mineral N in the soil, it may be more beneficial for legumes from low-nutrient ecosystems to take up N via its roots as discussed before [35]. This behaviour has been observed in white clover where N concentration was unaffected by P deficiency and N_2_ fixation increased under P deficiency by approximately 30% [47,48]. Contrary to that, PUpE was greater in the inoculated, well-irrigated plants, regardless of the species. The reduced P forced the plants to quickly acquire all available P to provide for the nodules formation, as the presence of the rhizobia will ensure extra N in the future [47,48]. In other studies, it was found that total root length, root surface area, root diameter, specific root length and root mass ratios were factors that contributed largely to high PUpE in legumes [26,49,50]. In this study, the four legumes had significantly lower root:shoot ratios when inoculated than when non-inoculated (Figure 2). This behavior is associated with the legumes capacity to fix N which alleviates N deficiency and reduces the investment into below-ground biomass [51,52].

No differences in PNUE were observed for any of the well-irrigated plants. *R. sphaerocarpa* and *E. barnadesii* were the ones with the greatest PPUE in well-irrigated conditions. Under this treatment, inoculation in the absence of P clearly enhanced PPUE. When plants are inoculated, they require additional nutrients for nodules formation. The inoculation in the absence of P might be the explanation for a quicker use of any molecule of P in the vicinity of the plants, thus aiding them to form nodules. The four species showed similar patterns of PPUE under drought stress. In fact, PPUE were at their maximum in the high P treatment, proving that plants would reduce energetic costs when mineral nutrition is available. Due to the low P, supply plants show N-induced demand for P [53], which is satisfied, allocating resources to the roots, the organs for nutrients acquisition as proven in this study (Figure 2) and as described for other legumes [41,53,54]. In our study, plants of the four species under water-stress benefitted from the presence of their rhizobia as all the inoculated ones enhanced their PNUE and PPUE, establishing a strong dependency on the symbionts to better acquire and accumulate resources.

The four species studied in this investigation survived better and produced more biomass when water and P were available. Their behavior can be related to the environments where they come from. *G. florida* and *E. barnadessi* are more efficient in the use of nutrients even in the more restrictive conditions of drought and low P, whereas *G. cinerea* and *R. sphaerocarpa* proved to be less efficient in the use of nutrients under stressful conditions. Despite these differences in the use of the available resources, the four species proved to be able to survive under stress, indicating that different strategies are needed to face drought and lack of nutrients in the very poor soils of the Iberian Peninsula where they originate. *R. sphaerocarpa* has a tap root that can access deep water in nature, thus being susceptible to the water scarcity conditions in this study. The other three species are more resistant to water stress and responded better to these conditions in our study by devoting more resources to roots. As described in other species [53,55], the presence of P in the growing media enhances plant biomass production. The simultaneous presence of elite symbionts enhances plant performance. To our knowledge, this is the first time that the responses of these four shrubby legumes have been investigated with regards to P availability in the soil. It is also the first time that it is reported how P and water stress plants overcome the lack of nutrients by enhancing biological nitrogen fixation by means of greater biomass allocation to roots and modifying the source of mineral nutrients towards biological nitrogen fixation. These findings highlight the importance of shrubby legumes in nature and how the correct selection of plants and their symbionts can be taken as an important tool to re-vegetate soils and to recover the functioning of natural ecosystems. Both the legume and rhizobial species have evolved in a given environment, being adapted to those soils conditions, which makes them ideal to be reintroduced in their natural environments, as they can cope with every environmental stress when properly reintroduced.

## Figures and Tables

**Table 1:** Relative growth rates of roots of *G. cinerea*, *G. florida*, *E. barnadesii* and *R. sphaerocarpa* plants supplied with high P (500 µM) and low P (5 µM) phosphate grown with and without rhizobial inoculation under two water regimes (water: well irrigated and drought: water stressed). Values are presented as means (*n* = 13–15) with standard deviation. The different letters indicate significant differences among the treatments, where the prime lettering indicates the comparisons between the same organ (*p* ≤ 0.05) and the second between plant species.

	Control	Inoculated	High P	Inoc + High P	Low P	Inoc + Low P
**Water**						
*G. cinerea*	51.38 ± 1.202 ^a,a^	133.74 ± 9.03 ^b,a^	63.34 ± 4.01 ^c,a^	55.97 ± 6.04 ^c,a^	117.85 ± 9.14 ^a,a^	171.60 ± 8.43 ^b,a^
*G. florida*	14.68 ± 1.86 ^a,b^	138.39 ± 16.49 ^b,b^	16.20 ± 1.66 ^b,b^	207.86 ± 12.29 ^c,b^	82.40 ± 4.11 ^a,b^	155.86 ± 12.77 ^b,b^
*R. sphaerocarpa*	8.60 ± 0.03 ^a,b^	56.78 ± 3.21 ^b,b^	62.99 ± 10.44 ^a,c^	136.97 ± 14.35 ^c,c^	60.26 ± 1.12 ^a,c^	90.23 ± 7.91 ^b,b^
*E. barnadesii*	76.12 ± 7.87 ^a,c^	142.71 ± 12.67 ^b,a^	9.99 ± 0.65 ^c,d^	39.50 ± 3.08 ^d,a^	5.66 ± 0.63 ^a,c^	172.86 ± 6.14 ^d,b^
**Drought**						
*G. cinerea*	0.41 ± 0.002 ^a,a^	1.06 ± 0.003 ^a,a^	0.40 ± 0.018 ^b,a^	0.74 ± 0.012 ^b,a^	0.94 ± 0.002 ^c,a^	1.36 ± 0.041 ^a,a^
*G. florida*	0.55 ± 0.07 ^a,b^	1.10 ± 0.003 ^b,b^	1.29 ± 0.055 ^c,b^	1.65 ± 0.038 ^c,b^	0.60 ± 0.017 ^a,b^	1.24 ± 0.055 ^b,b^
*R. sphaerocarpa*	0.07 ± 0.004 ^a,b^	0.45 ± 0.040 ^b,b^	0.50 ± 0.031 ^a,a^	1.09 ± 0.029 ^b,b^	0.48 ± 0.022 ^a,b^	0.72 ± 0.060 ^b,b^
*E. barnadesii*	0.60 ± 0.002 ^a,a^	1.13 ± 0.007 ^b,a^	0.12 ± 0.003 ^a,c^	0.15 ± 0.019 ^b,a^	0.71 ± 0.031 ^a,a^	1.37 ± 0.146 ^b,b^

## References

[1] Schimel D.S. (2010). Dry lands in the earth system. Science.

[2] Ruiz-Díez B., Fajardo S., Puertas-Mejía M.A., de Felipe M.R., Fernández-Pascual M. (2009). Stress tolerance, genetic analysis and symbiotic properties of root-nodulating bacteria isolated from Mediterranean leguminous shrubs in Central Spain. Arch. Microbiol..

[3] Fagg C.W., Stewart J.L. (1994). The value of Acacia and Prosopis in arid and semi-arid environments. J. Arid. Environ..

[4] Pérez-Fernández M.A., Calvo-Magro E., Valentine A. (2016). Benefits of the symbiotic association of shrubby legumes for the rehabilitation of degraded soils under Mediterranean Climatic conditions. Land Degrad. Dev..

[5] Stevens G.G., Pérez-Fernández M.A., Morcillo R.J.L., Kleinert A., Hills P., Brand D.J., Steenkamp E.T., Valentine A.J. (2019). Roots and Nodules Response Differently to P Starvation in the Mediterranean-Type Legume *Virgilia divaricata*. Front. Plant. Sci..

[6] Lal R. (2004). Soil carbon sequestration impacts on global climate change and food security. Science.

[7] Herrera M.A., Salamanca C.P., Barea J.M. (1993). Mycorrhizal fungi and rhizobia to recover desertified Mediterranean ecosystems. Appl. Environ. Microbiol..

[8] Rodríguez-Echeverría S., Pérez-Fernández M.A. (2003). Soil fertility and herb facilitation mediated by *Retama sphaerocarpa*. J. Veg. Sci..

[9] Magadlela A., Pérez-Fernández M.A., Kleinert A., Dreyer L.L., Valentine A.J. (2016). Source of inorganic N affects the cost of growth in a legume tree species (*Virgilia divaricata*) from the Mediterrean-type Fynbos ecosystem. J. Plant Ecol..

[10] Pérez-Fernández M.A., Calvo-Magro E., Rodríguez-Sánchez J., Valentine A. (2017). Differential growth costs and nitrogen fixation in *Cytisus multiflorus* (L’Hér.) Sweet and *Cytisus scoparius* (L.) Link are mediated by sources of inorganic N. Plant Biol..

[11] Vadez V., Lasso J.H., Beck D.P., Drevon J.J. (1999). Variability of N2-fixation in common bean (*Phaseolus vulgaris* L.) under P deficiency is related to P use efficiency. Euphytica.

[12] Hogh-Jensen H., Schjoerring J.K., Soussana J.F. (2002). The influence of phosphorus deficiency on growth and nitrogen fixation of white clover plants. Annal. Botany.

[13] Olivera M., Tejera N., Iribarne C., Ocana A., Lluch C. (2004). Growth, nitrogen fixation and ammonium assimilation in common bean (Phaseolus vulgaris): Effect of phosphorus. Physiol. Plant.

[14] Moro M.J., Domingo F., Bermudez-de-Castro F. (1992). Acetylene reduction activity (ARA) by the shrub legume *Adenocarpus decorticans* Boiss. in southern Spain (Almeria). Acta Oecol..

[15] Streeter J.G. (2003). Effects of drought on nitrogen fixation in soybean root nodules. Plant Cell Environ..

[16] Wadisirisuk P., Danso S.K.A., Hardarson G., Bowen G.D. (1989). Influence of Bradyrhizobium japonicum location and movement on nodulation and nitrogen fixation in soybeans. Appl. Environ. Microbiol..

[17] Shamseldin A., Moawad H. (2010). Inhibition of nitrogenase enzyme and completely suppression of nodulation in common bean (Phaseolus vulgaris L.) at high level of available nitrogen. Am.-Eurasian J. Agric. Environ. Sci..

[18] Venkateswarlu B., Rao A.V., Lahiri A.N. (1983). Effect of water stress on nodulation and nitrogenase activity of guar (*Cyamopsis tetragonoloba* (L.) Taub.). Proc. Indian Acad. Sci. Plant. Sci. USA.

[19] Abberton M.T., MacDuff J.H., Vagg S.A.H., Marshall A.H., Michaelson-Yeates T.P.T. (2000). Nitrogen Fixation in Hybrids of White Clover (*Trifolium repens* L.) and Caucasian Clover (*Trifolium ambiguum* M. Bieb). J. Agron. Crop. Sci..

[20] Sengupta D., Kannan M., Reddy A.R. (2011). A root proteomics-based insight reveals dynamic regulation of root proteins under progressive drought stress and recovery in Vigna radiata (L.) Wilczek. Planta.

[21] López-González G. (2007). Guía de los árboles y arbustos de la Península Ibérica y Baleares (Especies silvestres y las cultivadas más comunes).

[22] Hoagland D.R., Arnon D.I. (1950). The Water-Culture Method of Growing Plants Without Soil.

[23] Ogbaga C.C., Stepien P., Johnson G.N. (2014). Sorghum (*Sorghum bicolor*) varieties adopt strongly contrasting strategies in response to drought. Physiol. Plant.

[24] Lachica M., Aguilar A., Yánez J. (1973). Análisis foliar. Métodos utilizados en la Estación Experimental del Zaidín CSIC (II). An. Edaf. Agrobiol.

[25] Bouat A., Crouzet C. (1965). Notes techniques sur un appareil semiautomatique de clorage de l’azote et de certains composes volatiles. Ann. Agric..

[26] Capitan F., Martínez F. (1954). Sobre la determinación espectrofotométrica de fósforo con amidol. An. Edaf. Agribiol.

[27] Hammond J.P., Broadley M.R., White P.J., King G.J., Bowen H.C., Hayden R., Meacham M.C., Mead A., Overs T., Spracklen W.P. (2009). Shoot yield drives phosphorus use efficiency in *Brassica oleracea* and correlates with root architecture traits. J. Exp. Bot..

[28] Bazzaz F.A., Grace J., Bazzaz F.A., Grace J. (1987). Allocation of Resources in Plants: State of Science and Critical Questions. Plant Resource Allocation.

[29] Ndzwanana Z., Tsvuura Z., Valentine A.J., Pérez-Fernández M.A., Magadlela A. (2019). Differential patterns of nitrogen nutrition and growth cost of the indigenous *Vachellia sieberiana* and the introduced *Chromolaena odorata* in the savannah environment. AOB Plants.

[30] Míguez-Montero M.A., Valentine A., Pérez-Fernández M.A. (2019). Regulatory effect of phosphorus and nitrogen on nodulation and plant performance of leguminous shruas. AoB Plants.

[31] Akinrade E.A., Iroh L., Obigbesan G.O. (2006). Differential expression of alu- miniumtolerance mechanisms in cowpea genotypes under phosphorus limitation. J. Appl. Sci..

[32] Tian Q.Y., Chen F.J., Liu J.X., Zhang F.S., Mi G.H. (2008). Inhibition of maize root growth by high nitrate supply is correlated with reduced IAA levels in roots. Plant Physiol..

[33] López-Pintor A., Gómez Sal A., Rey Benayas J.M. (2006). Shrubs as a source of spatial heterogeneity—The case of *Retama sphaerocarpa* in Mediterranean pastures of central Spain. Acta Oecol..

[34] Pérez-Fernández M.A., Calvo-Magro E., Ramírez-Rojas I., Moreno Gallardo L., Valentine A. (2016). Patterns of growth costs and nitrogen acquisition in *Cytisus striatus* (Hill)Rothm, and *Cytisus balansae* (Boiss.) Ball are mediated by sources of inorganic N. Plants.

[35] Kaschuk G., Xinyou Y., Hungria M., Leffelaar P., Giller K.E., Kuyper W.T. (2012). Photosynthetic adaptation of soy bean due to varying effectiveness of N_2_ fixation by two distinct *Bradyrhizobium japonicum* strains. Environ. Exp. Bot..

[36] Magadlela A., Steenkamp E.T., Valentine A.J. (2015). Variable P supply affect N metabolism in a legume tree, *Virgilia divaricata*, from nutrient-poor Mediterranean-type ecosystems. Funct. Plant Biol..

[37] Bedoussac L., Journet E.P., Hauggaard-Nielsen H., Naudin C., Corre-Hellou G., Jensen E.S., Prieur L., Justes E. (2015). Ecological principles underlying the increase of productivity achieved by cereal-grain legume intercrops in organic farming: a review. Agron. Sustain. Dev..

[38] Valentine A.J., Kleinert A., Benedito V.A. (2017). Adaptive strategies for nitrogen metabolism in phosphate deficient legume nodules. Plant Sci..

[39] Parker M.A., Wanda M., Parker I.M. (2006). Growth of an invasive legume is symbiont limited in newly occupied habitats. Divers. Distrib..

[40] Pérez-Fernández M.A., Lamont B.B. (2016). Competition and facilitation between Australian and Spanish legumes in seven Australian soils. Plant Species Biol..

[41] Sprent J.I., Gehlot H.S. (2010). Nodulated legumes in arid and semi-arid environments: Are they important?. Plant Ecol. Divers..

[42] Lü X.T., Reed S., Yu Q., He N.P., Wang Z.W., Han X.G. (2013). Convergent responses of nitrogen and phosphorus resorption to nitrogen inputs in a semiarid grassland. Glob. Chang. Biol..

[43] Hardarson G., Danso S.K.A. (1993). Methods for measuring biological nitrogen fixation in grain legumes. Plant Soil.

[44] Unkovich M., Herridge D., Peoples M., Cdisch G., Boddey B., Giller K., Alves B., Chalk P. (2008). Measuring Plant-Associated Nitrogen Fixation in Agricultural Systems.

[45] Vardien W., Valentine A.J., Mesjasz-Przybyłowicz J., Przybyłowicz W.J., Wang Y., Steenkamp E.T. (2014). Nodules from Fynbos legume *Virgilia divaricata* have high functional plasticity under variable P supply levels. J. Plant Physiol..

[46] Magadlela A., Kleinert A., Dreyer L.L., Valentine A.J. (2014). Low phosphorus conditions affect the nitrogen nutrition and associated carbon costs of two legume tree species from a Mediterranean-type ecosystem. Aust. J. Bot..

[47] Tsvetkova G.E., Georgiev G.I. (2007). Changes in phosphate fractions extracted from different organs of phosphorus starved nitrogen fixing pea plants. J. Plant Nutr..

[48] Maxwell T.M.R., Moir J.L., Edwards G.R. (2013). Phosphorus response and efficiency of four adventive annual clovers grown in a New Zealand high country soil under glasshouse conditions. New Zeal. J. Agric. Res..

[49] Almeida J.P.F., Hartwig U.A., Frehner M., Nösberger J., Lüscher A. (2000). Evidence that P deficiency induces N feedback regulation of symbiotic N2 fixation in white clover (*Trifolium repens*). J. Exp. Bot..

[50] Fageria N.K., Moreira A., Moraes L.A.C., Moraes M.F. (2014). Root growth, nutrient uptake, and nutrient-use efficiency by roots of tropical legume cover crops as influenced by phosphorus fertilization. Commun. Soil. Sci. Plant.

[51] Pang J., Tibbett M., Denton M.D., Lambers H., Siddique K.H.M., Bolland M.D.A., Revell C.K., Ryan K.H. (2010). Variation in seedling growth of 11 perennial legumes in response to phosphorus supply. Plant Soil.

[52] Markham J.H., Zekveld C. (2007). Nitrogen fixation makes biomass allocation to roots independent of soil nitrogen supply. Can. J. Bot..

[53] Power S.C., Cramer M.D., Verboom G.A., Chimphango S.B.M. (2010). Does phosphate acquisition constrain legume persistence in the fynbos of the Cape Floristic Region?. Plant Soil.

[54] Maistry P.M., Muasya A.M., Valentine A.J., Chimphango S.B.M. (2015). Increasing nitrogen supply stimulates phosphorus acquisition mechanisms in the fynbos species Aspalathus linearis. Funct. Plant Biol..

[55] MacAlister D., Muthama Muasya A., Chimphango S.M. (2018). Linking root traits to superior phosphorus uptake and utilization efficiency in three Fabales in the Core Cape Subregion, South Africa. Funct. Plant Biol..

